# Novel betaherpesviruses and gammaherpesviruses in bats from central China

**DOI:** 10.1038/s41598-024-61290-1

**Published:** 2024-05-09

**Authors:** Shuhui Duan, Zemin Li, Xu Zhang, Xue-Jie Yu

**Affiliations:** State Key Laboratory of Virology, School of Public Health, Wuhan University, Wuhan, 430071 People’s Republic of China

**Keywords:** Gammaherpesvirus, Betaherpesvirus, *Myotis davidii*, *Rhinolophus pusillus*, Bat, Phylogenetics, Virology

## Abstract

Herpesviruses are large double-stranded DNA viruses that cause infections in animals and humans with a characteristic of latent infectious within specific tissues. Bats are natural hosts of variety human-infecting viruses and recently have been described as hosts for herpesviruses in several countries around the world. In this study we collected 140 insectivorous bats in the neighboring urban areas of Wuhan City, Hubei Province in the central China between 2020 and 2021. Nested PCR targeting the *dpol* gene sequence indicated that a total of 22 individuals (15.7% of the sample) tested positive for herpesvirus with 4 strains belonging to the genus *Betaherpesvirus* and the remaining 18 strains classified as *Gammahersvirus*. Furthermore, the herpesvirus prevalence in *Rhinolophus pusillus* was higher at 26.3%, compared to 8.4% in *Myotis davidii*. The RP701 strain from *R. pusillus* was the predominant gammaherpesvirus strain detected in bats, accounting for 94.4% (17/18) of all strains. The variations in γ-herpesviruses genomic sequences was evident in phylogenetic tree, where RP701 strain was clustered together with ruminant γ-herpesviruses, while MD704 strain formed a distinct clade with a hedgehog γ-herpesvirus. Four betaherpesviruses exclusively identified from *M. davidii*, with nucleotide identities ranging from 79.7 to 82.6% compared to known betaherpesviruses. Our study provided evidence that *M. davidii* can sever as natural host for β-herpesviruses, which extended the host species range. In conclusion, we found that bats from central China harbored novel β-herpesviruses and γ-herpesviruses which were phylogenetically related to ruminant γ-herpesvirus and hedgehog γ-herpesvirus. Our study indicates that bats are natural hosts of β- and γ-herpesviruses and further studies are needed to determine whether there is cross-species transmission of herpesviruses between bats and other animals, or humans.

## Introduction

Outbreaks of emerging zoonotic diseases have posed significant threats to public health and caused substantial disruptions to the global economy in recent years. Due to their widely distribution and unique social habitats, bats serve as a reservoir for a large variety of pathogens. Cross-species transmission of bat-borne viruses such as SARS-CoV, lyssaviruses, filoviruses, henipaviruses, and Ebola viruses^1–9^ pose significant threats to both livestock health and human well-being.

Herpesviruses, belonging to the family *Herpesviridae*, are enveloped viruses with a linear double-stranded DNA genome ranging from 124 to 295 kbp. Herpesviruses have been found in mammals, birds, reptiles, amphibians, fishes, and mollusks^10,11^. All herpesviruses are able to remain latent infection in their natural hosts^12,13^. Based on biological properties and genome sequence similarities, mammal herpesviruses are classified into three subfamilies, namely *Alpha(α)herpesvirinae*, *Beta(β)herpesvirinae*, and *Gamma(γ)herpesvirinae*, along with a total of 17 genera. The *Gammaherpesvirinae* subfamily is further divided into *Macavirus*, *Percavirus*, *Lymphocryptovirus*, *Bossavirus*, *Manticavirus*, *Patagivirus*, and *Rhadinovirus* genera^11,14^. The *Betaherpesvirinae* is divided into five genera: *Cytomegalovirus*, *Muromegalovirus*, *Proboscivirus*, *Roseolovirus*, and *Quwivirus*^14^. Betaherpesviruses differ from alpha- and gammaherpesviruses in their restricted host range and long infection cycle. Herpesviruses can cause significant human diseases. The human β-herpesvirus, including human cytomegalovirus (HCMV), human herpesvirus (HHV) 6A, HHV-6B, and HHV-7, can cause severe diseases including encephalitis and cognitive decline in immune-compromised and immune-naive populations^15,16^. Besides, γ-herpesvirus mainly targeted lymphoid cell lineage causing neoplasias, including human γ-herpesvirus 4 (also known as Epstein-Barr virus) and human γ-herpesvirus 8 (also known as Kaposi’s sarcoma-associated herpesvirus)^17^.

The first study of bat herpesviruses dated back to 1996, in which Tandler described cytomegalovirus-like particles in salivary glands of the little brown bat (*Myotis lucifugus*) by light and electron microscopy^18^. It was not until 2007 that the sequence of the bat herpesvirus was initially characterized^12^. Since then, many bat herpesviruses sequences have been obtained from diverse bat species worldwide^19–24^. To date, evidences existed that bat *γ*-herpesviruses, such as the *Myotis* γ-herpesvirus 8 (BGHV8), could replicate and cause cytopathic effects in bat and other mammalian cells^25^. The replication of the bat β-herpesvirus was restricted in specific types of cells like kidney cells^22^.

In this study, we investigated herpesviruses in insectivorous bats collected from Hubei Province in the central China. Upon identifying the herpesviruses, subsequent phylogenetic analyses of the sequences obtained were conducted to unravel epidemiological characteristics.

## Materials and methods

### Bat collection and bat DNA extraction

Bats were collected from Karst caves in the neighboring urban areas of Wuhan City, including Jingzhou and Xianning, Hubei Province in central China from 2020 to 2021 as described previously^26^. Bat species were initially identified morphologically^27^ and the accuracy of species identification was subsequently confirmed by DNA sequencing the PCR amplified cytochrome b (*cytB*) genes of representative bats of each species^28^.

Genomic DNA was extracted from intestine and liver tissues of each individual bat using DNA extraction kits (Qiagen, Valencia, CA). The DNA extraction process was carried out according to the manufacturer’s instructions. The experimental procedure could be briefly as follows: after thorough homogenizing the tissue samples, proteinase K and buffer ATL were successively added into the mixture, followed by digestion and incubation processes. Subsequently, the resultant mixture was transferred into a spin column for centrifugation, obtaining a solution containing the genomic DNA. The amount and purity of the DNA were estimated using NanoDrop One equipment (Thermo Scientific, Rockford, CA) and stored at −20 ºC until use.

### Detection of bat herpesviruses

Nested PCR assay was performed with degenerate primers targeting herpesviruses DNA polymerase gene (*dpol*) (approximately 200 bp) for molecular herpesvirus identification^29^. Further steps were taken employing purpose-designed primers (Round 1: dpol-F11 5′-CGCTAATGAGCTGGCACAAG-3′, dpol-F12 5′-CKSCKWAGACARTCWCCACA-3′, dpol-R11 5′-GAGATGGTCATGTGTGGCGG-3′. Round 2: dpol-F21 5′- HGGGTCTGGRTASGGMARR-3′, dpol-R21 5′-CAGGCTGTTAGTGCCAATGT-3′). To further characterize the herpesviruses, nested PCR targeting 500 bp herpesvirus glycoprotein B gene (*gB*) were carried out^30^. PCR products were electrophoresed and purified from 1.5% agarose gels with a gel extraction kit (Tsingke Biotechnology, Beijing, China). The purified PCR products were sequenced bidirectionally using Sanger sequencing. The 5’- and 3’- ends of the sequences derived from primers were trimmed.

### Phylogenetic analysis

A BLAST search was conducted (https://blast.ncbi.nlm.nih.gov/Blast.cgi), and the most similar herpesvirus sequences were aligned using the ClustalW algorithm through MEGA X software. Nucleotide sequences were subjected to phylogenetic analysis. The phylogenetic trees were constructed using the Maximum Likelihood (ML) method of the Kimura 2-parameter model in MEGA X^31^. The bootstrap method (1,000 replicates) was applied to assess the reliability of the tree.

### Ethical statement and permits

The study was conducted with the approval of the Ethics Committee of the Medical School, Wuhan University (WHU2020-YF0023), and was in accordance with the ARRIVE guidelines (https://arriveguidelines.org). We confirm that all methods were performed in accordance with the relevant guidelines and regulations. All efforts were made to minimize discomfort to the animals.

## Results

### Prevalence of herpesvirus in bats

During the period of 2021 to 2022, a total of 140 bats were sampled from the neighboring areas around Wuhan City, including Xianning and Jingzhou, in Hubei Province, China. We investigated the prevalence of herpesvirus in tissue samples (liver and intestines) collected from these insectivorous bats (Table 1), which consisted of two different species, *Myotis davidii* (n = 83, 59.3%) and *Rhinolophus pusillus* (n = 57, 40.7%). Within Xianning District, 121 bats specimens were collected with *M. davidii* constituting 52.9% and *R. pusillus* accounting for 47.1%. The remaining 19 individuals of *M. davidii* originated from Jingzhou District.

**Table 1:** Summary of bat sampling information.

Sampling date	Sampling area	Bat family	Bat species	Bat No	HV positive (%)
July 2020	Xianning	*Vespertilionidae*	*M. davidii*	64	6 (9.4%)
		*Rhinolophidae*	*R. pusillus*	57	15 (26.3%)
July 2021	Jingzhou	*Vespertilionidae*	*M. davidii*	19	1 (5.3%)
Total				140	22 (15.7%)

Nested PCR with *dpol* primers showed that 15.7% (22/140) bats were herpesvirus positive, including 8.4% (7/83) *Myotis davidii* and 26.3% (15/57) *Rhinolophus pusillus* (Table 1). These herpesviruses were further classified into *γ*-herpesviruses (81.8%, 18/22) and β-herpesviruses (18.2%, 4/22) (Table 2). Among all samples obtained from *M. davidii* tested positive for herpesvirus, there was only one specimen (MD704) originated from Jingzhou District.

**Table 2:** Herpesviruses identified in bats with degenerate primers of *dpol* and *gB* gene using nested PCR.

**HV-positive bat number**	Bat species	Tissue	Herpesvirus	Accession number of closest match in GenBank		Accession number of this study
				Nucleotide identity (%)	Amino acid identity (%)	
PCR results with *dpol* primers						
RP739	*R. pusillus*	Intestine	γ-herpesvirus	KR261894 (100%)	ALH21052 (100%)	OP793818
RP746	*R. pusillus*		γ-herpesvirus	KR261894 (100%)	ALH21052 (100%)	OP793819
RP752	*R. pusillus*		γ-herpesvirus	KR261894 (100%)	ALH21052 (100%)	OP793817
MD751	*M. davidii*		β-herpesvirus	KR608285 (81.6%)	AMY98774 (78.7%)	OP793835
MD761	*M. davidii*		β-herpesvirus	KR608285 (79.7%)	AMY98774 (85.4%)	OP793838
MD773	*M. davidii*		β-herpesvirus	KR608285 (82.6%)	AMY98774 (80.8%)	OP793836
MD779	*M. davidii*		β-herpesvirus	KR608285 (81.6%)	AMY98774 (79.6%)	OP793837
MD704	*M. davidii*	Liver	γ-herpesvirus	MF579869 (75.2%)	ATU31556 (82.6%)	OP793820
RP707	*R. pusillus*		γ-herpesvirus	KR261894 (99.4%)	ALH21052 (98.0%)	OP793821
RP701	*R. pusillus*		γ-herpesvirus	KR261894 (100%)	ALH21052 (100.0%)	OP793825
RP705	*R. pusillus*		γ-herpesvirus	KR261894 (100%)	ALH21052 (100.0%)	OP793823
RP712	*R. pusillus*		γ-herpesvirus	KR261894 (100%)	ALH21052 (100.0%)	OP793826
RP716	*R. pusillus*		γ-herpesvirus	KR261894 (100%)	ALH21052 (100.0%)	OP793830
RP739	*R. pusillus*		γ-herpesvirus	KR261894 (100%)	ALH21052 (100.0%)	OP793818
RP752	*R. pusillus*		γ-herpesvirus	KR261894 (100%)	ALH21052 (100.0%)	OP793817
RP763	*R. pusillus*		γ-herpesvirus	KR261894 (100%)	ALH21052 (100.0%)	OP793833
RP765	*R. pusillus*		γ-herpesvirus	KR261894 (100%)	ALH21052 (100.0%)	OP793829
RP806	*R. pusillus*		γ-herpesvirus	KR261894 (100%)	ALH21052 (100.0%)	OP793832
RP815	*R. pusillus*		γ-herpesvirus	KR261894 (100%)	ALH21052 (100.0%)	OP793831
RP816	*R. pusillus*		γ-herpesvirus	KR261894 (100%)	ALH21052 (100.0%)	OP793824
RP817	*R. pusillus*		γ-herpesvirus	KR261894 (100%)	ALH21052 (100.0%)	OP793822
RP818	*R. pusillus*		γ-herpesvirus	KR261894 (100%)	ALH21052 (100.0%)	OP793828
MD727	*M. davidii*		γ-herpesvirus	KR261894 (100%)	ALH21052 (100.0%)	OP793834
MD744	*M. davidii*		γ-herpesvirus	KR261894 (100%)	ALH21052 (100.0%)	OP793827
PCR results with *gB* primers						
RP701	*R. pusillus*	Liver	γ-herpesvirus	KR261912 (98.6%)	ALH21101 (100.0%)	OP793839
RP716	*R. pusillus*		γ-herpesvirus	KR261912 (98.8%)	ALH21101 (100.0%)	OP793840

Seven partial *dpol* gene sequences of herpesviruses were obtained from intestine samples. The sequences were analyzed using the BLASTN and BLASTX algorithms of the NCBI database. Out of the seven bats that tested positive for herpesvirus, three individuals were identified as *R. pusillus*, whereas the other four were *M. davidii.* BLAST analysis of three *dpol* gene sequences from *R. pusillus* revealed that these sequences were 100% identical to each other, and were classified as the subfamily *Gammaherpesvirinae*. Interestingly, these sequences were identical to a γ-herpesvirus RB/13YF97 from bat *R. blythi* in Guangdong Province, which is located in southern China (GenBank: KR261894)^32^. The other four sequences were obtained from *M. davidii* with nucleotide identity of > 97% to each other and belonged to the subfamily *Betaherpesvirinae*. They were most identical to *M. emarginatus* β-herpesvirus 1 detected in Spain (GenBank: KR608285) with nucleotide identity ranged from 79.7 to 82.6%, showing 69.2 to 82.7% amino acid identity with corresponding protein sequences.

Seventeen partial *dpol* gene sequences were obtained from bat liver specimens, which belonged to the subfamily *Gammaherpesvirinae*. According to the pairwise alignment between these sequences, it showed that MD704 strain detected in *M. davidii* from Jingzhou District shared approximately 80% nucleotide identity with other 16 sequences. MD704 was most identical to the BtHVNeoV4 (GenBank: MF579869) from South Africa, showing 75.2% identity at the nucleotide level and 82.6% amino acid identity, respectively. The remaining 16 *dpol* gene sequences represented by RP701 was almost identical with each other (nucleotide identities: 99.3%–100%), indicating these bats were infected with the same viral strain. At the same time, this strain was most identical to the γ-herpesvirus RB/13YF97^32^ (nucleotide identities > 99%). Among these bats, herpesviruses were both identified in the intestine and liver tissues of only two specific individuals, namely RP739 and RP752. In this study, β-herpesviruses obtained only from the intestine tissues of *M. davidii*.

For samples positive for the *dpol* gene, we proceeded to amplify the herpesvirus glycoprotein B gene (*gB*) using nested PCR. The results showed that only two samples (RP701 and RP716) were PCR positive for *gB* gene (Table 2). BLAST analysis of the *gB* nucleotide sequences showed that they were almost identical with each other (identity > 99%) and mostly identical (> 98%) to a herpesvirus from the lesser Asiatic yellow house bat in China (GenBank: KR261912).

### Phylogenetic analyses

**Figure 1:**
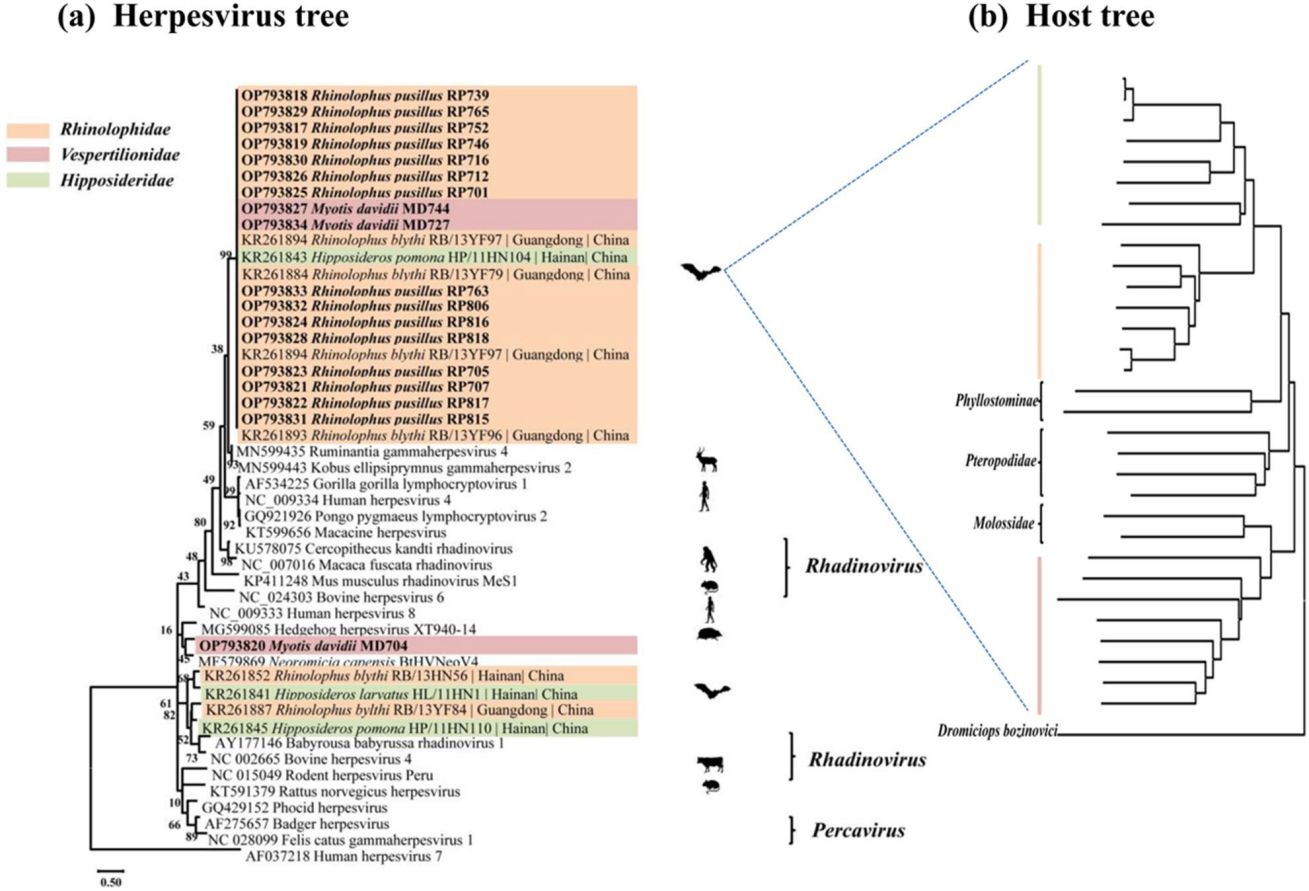
Phylogenetic trees of Gammaherpesvirus and bats. The tree was constructed based on the *dpol* gene sequences (160 bp) with 30 representatives of γ-herpesviruses from the GenBank database using the Maximum Likelihood method with 1,000 bootstrap repetitions. Human herpesvirus 7 served as an outgroup. Sequences generated in this study were highlighted in bold (**a**). The phylogeny of host bats was derived from the *cytB* sequences (1140 bp) of 31 bats from 6 bat families (*Hipposideridae*, *Rhinolophidae*, *Phyllostominae*, *Pteropodidae*, *Molossidae*, and *Vespertilionidae*) with *Dromiciops bozinovici cytB* sequences as an outgroup (**b**). Silhouette images were downloaded from PhyloPic (http://phylopic.org), an open-access database that stores reusable silhouette images of organisms.

Phylogenetic analysis based on *dpol* gene sequences showed that γ-herpesviruses from bats in this study were divided into two distinct clades (Fig. 1). Seventeen strains of γ-herpesviruses from this study formed a clade with five known bat γ-herpesviruses from Guangdong Province and Hainan Province, which are located in southern China. It was evident that the γ-herpesviruses were clustered within a same clade, while their corresponding hosts were from different bat species (*Rhinolophidae*, *Vespertilionidae* and *Hipposideridae*) widely distributed across the host phylogenetic tree (Fig. 1b). Additionally, these bat-borne γ-herpesviruses exhibited close evolutionary relationship with ruminant herpesviruses based on the phylogenetic tree (Fig. 1a). It indicated that bat herpesviruses potentially shared a common evolutionary ancestor with herpesviruses from other species, specifically ruminants. MD704 was distinct from all other bat γ-herpesviruses and independently formed a clade with a hedgehog herpesvirus with approximately 73% nucleotide identity. In general, the branches of bat γ-herpesviruses were cross-distributed with human γ-herpesviruses, ruminant γ-herpesviruses, and rodent γ-herpesviruses, indicating the interactive evolution of these γ-herpesviruses among different species.

**Figure 2:**
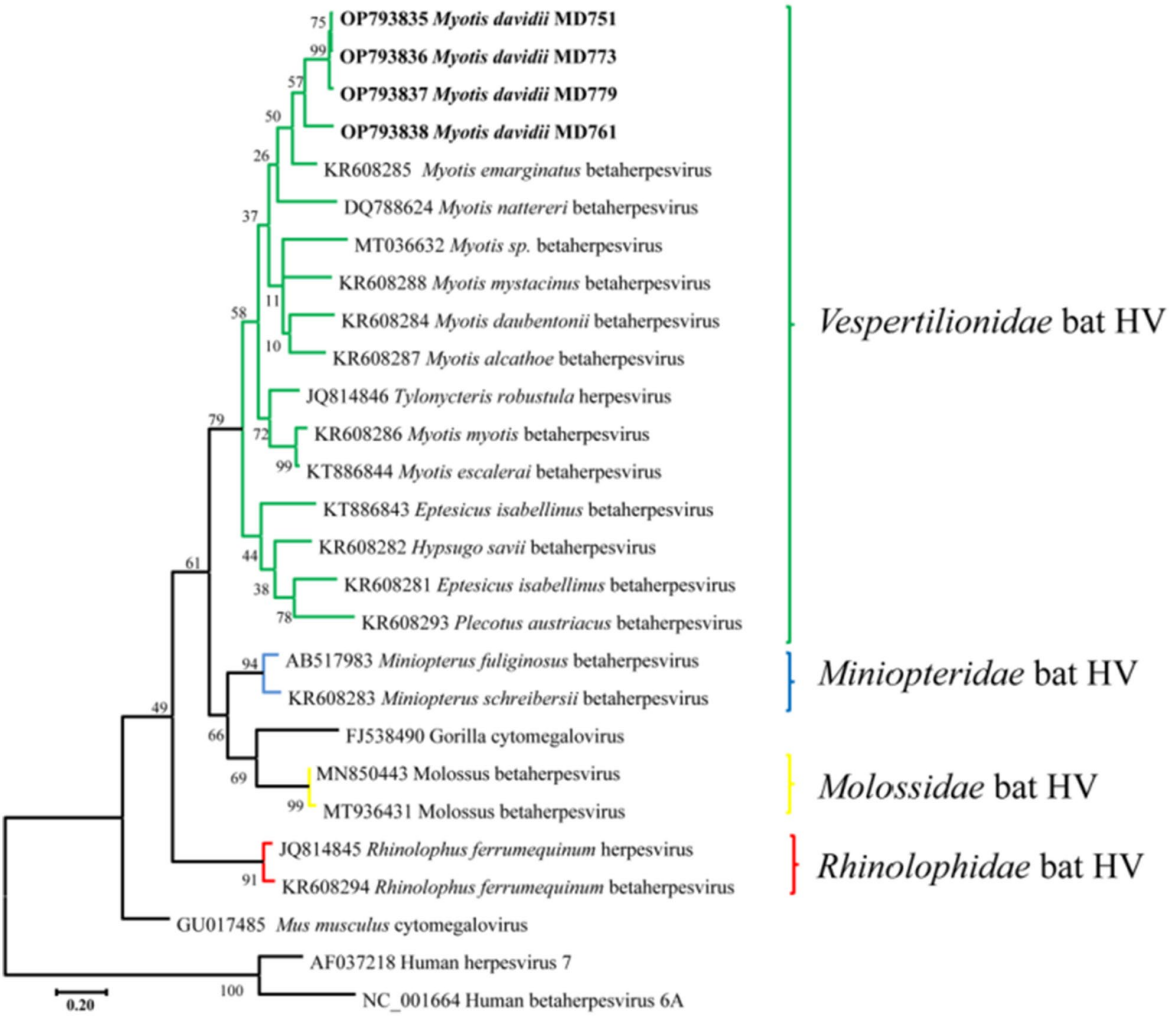
Betaherpesvirus phylogenetic tree based on partial *dpol* gene sequences. The virus detected in this study was highlighted in bold. The tree was constructed using the Maximum Likelihood method with 1000 bootstrap repetitions. Human herpesvirus 7 and human betaherpesvirus 6A served as outgroups.

In contrast to the γ-herpesviruses, four β-herpesviruses identified from *M. davidii* were clearly distinct from currently known *Vespertilionidae* bat β-herpesviruses. Three of four β-herpesviruses displayed a nucleotide identity approximately 98% and formed a clade with higher bootstrap support (Fig. 2). In addition, the cluster of *Vespertilionidae* bat HV included 2 different clades, one clade mainly containing sequences derived from *Myotis* sp. and the other one containing viral sequences detected in other *Vespertilionidae* genera.

**Figure 3:**
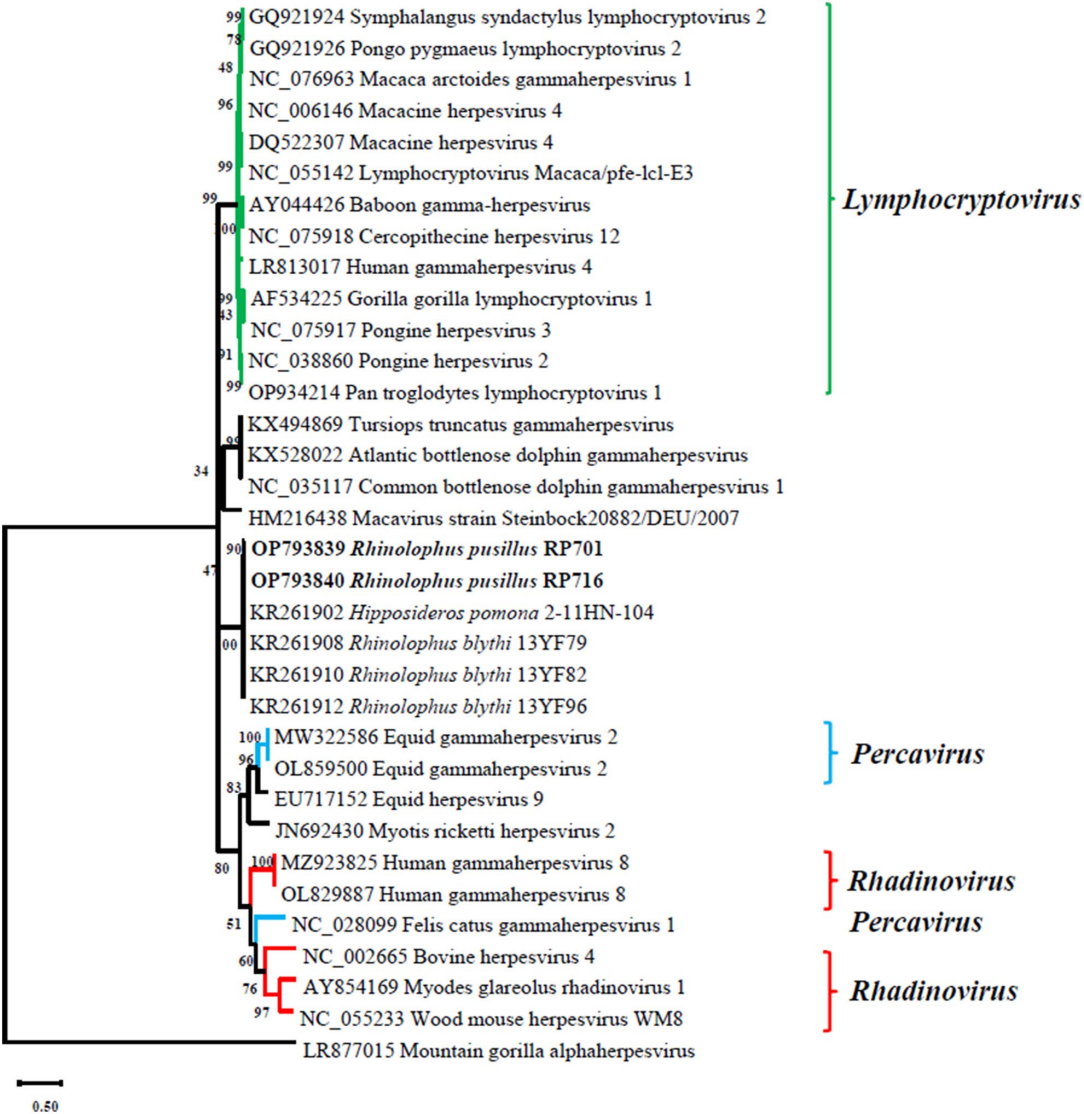
Phylogenetic analysis based on partial *gB* gene sequences (150 bp) of Gammaherpesvirus from bats. The virus detected in this study was highlighted in bold. The tree was constructed using the Maximum Likelihood method with 1000 bootstrap repetitions. Mountain gorilla alphaherpesvirus served as an outgroup.

We further analyzed the phylogeny of the bat γ-herpesviruses based on the *gB* gene sequences (Fig. 3). The results showed that the *gB* gene sequences of two γ-herpesviruses (RP701 and RP716) belonged to the genus *Lymphocryptovirus*, clustered with other known *Rhinolophidae* and *Hipposideridae* bat herpesviruses. In addition, these herpesviruses exhibited a close evolutionary relationship as evidenced by the high bootstrap value. Through comparison examination of their *gB* and *dpol* sequences, as well as analysis of the phylogenetic trees, it was evident that the two viral strains (RP701 and RP716) were almost identical, strongly suggesting that these two bats were likely infected by the same viral strain.

## Discussion

In this study, we detected herpesvirus *dpol* gene sequences in *M. davidii* and *R. pusillus* bats collected from neighboring areas around Wuhan City in central China. Nested PCR methods targeting well-conserved genes, such as *dpol* gene, were designed in order to study the bat-borne herpesviruses. Conserved genes exhibit a high degree of sequence similarity among different viral strains or species. By focusing on these genes, the PCR assays can detect a broad range of relative herpesviruses, increasing the likelihood of identifying known and potentially novel viral strains infecting bats from the central China. Previous studies have reported herpesviruses identified in bats which predominantly inhabit in southern regions of China such as Hainan and Guangdong^32^, and in the northern regions such as Beijing^20^, whereas there is limited reporting on their presence in the central China. Our study has verified the existence of multiple γ-herpesvirus and β-herpesviruses in the bat population in the central regions. It extended the geographic distribution of bat herpesviruses.

Currently, *Myotis davidii* is a predominant bat species in China, with its distribution spanning from central to northern of the country^33^. The least horseshoe bat, *R. pusillus* is widely distributed throughout the Indomalayan realm^34^. The prevalence of γ-herpesviruses in *M. davidii* (3.6%, 3/83) was lower than that in *R. pusillus* (21.7%, 15/57) based on *dpol* gene sequences in this study. Meanwhile, the β-herpesviruses were only found in *M. davidii*. It indicated that the prevalence and species of herpesviruses in bats might be related to the bat species genetic and habitat. The discovery of γ-herpesviruses and β-herpesviruses in *M. davidii* and *R. pusillus* extended the host species of bat herpesvirus.

According to the results of pairwise alignment and phylogenetic analysis of γ-herpesviruses *dpol* gene sequences, RP701 strain was the major epidemic γ-herpesvirus strain in the central regions, which constituted 94.4% of γ-herpesvirus strains identified in bats in this study. In addition, RP701 was identical to a bat γ-herpesvirus reported previously in Guangdong Province and Hainan Province in southern China^32^, indicating RP701 has a broad distribution from central China to southern China. It was evident that γ-herpesvirus RP701 strain widely circulating among bat population in the country clustered with ruminant herpesvirus, suggesting these viruses shared a common evolutionary ancestor. By comparing the genomic sequence of MD704 strain and analysis of the phylogenetic tree, it was found that the *dpol* gene sequence exhibited lower identity with other γ-herpesvirus sequences in the study. The reason for this outcome might be attributed to the different geographic locations and the host species. Each virus in the family *Herpesviridae* had a restricted host range^35^, while the branches of bat γ-herpesviruses were distributed among viral sequences from various species. It provided strong indirect evidence supporting that these herpesviruses might have undergo cross-species transmission.

The viral sequences of β-herpesviruses derived from *M. davidii* were confirmed in the same clade with other herpesviruses from different *Vespertilionidae* bats based on the phylogenetic analysis. It could be explained by the fact that these novel β-herpesviruses had more than one primary host among the *Vespertilionidae*, probably caused by close inter-species contact in roosts. Distinct from γ-herpesvirus, β-herpesviruses exhibited a relatively restricted host range, demonstrating a degree of host specificity.

In an extensive comparative analysis of herpesvirus in different host tissues, it revealed that there were organ tropisms for bat herpesvirus. Notably, β-herpesvirus nucleotide sequences were mainly obtained from the intestine tissues. In the case of different tissue samples from the same individual bat, viral sequences could be obtained in liver tissues, but not in the intestinal tissues, and the vice versa. It might stem from two main causes: Firstly, the outcome of detection could be influenced by the methods of sampling and laboratory procedure, such as the site of tissue sampling, storage conditions, and the sensitivity of the detection techniques applied. Secondly, disparities in the distribution and replication dynamics of the virus within different tissues can lead to substantial variations in viral loads among these tissues, meaning that in certain tissues with higher viral loads, detection is more feasible, while in those with lower viral loads, detecting the virus presents greater difficulty.

For samples that tested positive for the *dpol* gene, we attempted to obtain the corresponding *gB* gene segments but was only able to successfully obtain two *gB* gene sequences among the 22 positive samples. It illustrated that the *gB* gene segments displayed lower sensitivity compared to the *dpol* gene during the detection process. In addition, co-infections with beta- or gammaherpesviruses were not obtained in this study, but have been reported in primates^36^. Due to the limitation of sample size, the positive rate and species of bat herpesvirus might be different from the real situation.

In conclusion, we have found that bats from central China harbored β-herpesviruses and γ-herpesviruses similar to ruminant γ-herpesvirus and hedgehog γ-herpesvirus.

## Data Availability

These sequences of bat β-herpesviruses and γ-herpesviruses obtained in this study were deposited in the GenBank with accession numbers from OP793817 to OP793840. Additional data and information are available from the corresponding author on reasonable request.
